# A single‐cell transcriptomic atlas characterizes cell types and their molecular features in yak ovarian cortex

**DOI:** 10.1096/fj.202201176RR

**Published:** 2022-12-17

**Authors:** Jie Pei, Lin Xiong, Shaoke Guo, Xingdong Wang, Pengjia Bao, Xiaoyun Wu, Ping Yan, Xian Guo

**Affiliations:** ^1^ Key Laboratory of Yak Breeding Engineering of Gansu Province Lanzhou Institute of Husbandry and Pharmaceutical Sciences, Chinese Academy of Agricultural Sciences Lanzhou China; ^2^ Key Laboratory of Animal Genetics and Breeding on Tibetan Plateau Ministry of Agriculture and Rural Affairs Lanzhou China

**Keywords:** cortex, oocyte, ovary, scRNA‐seq, transcriptome, yak

## Abstract

The ovary as one of the most dynamic organs produces steroids to orchestrate female secondary sexual characteristics, harbors ovarian reserve for oocytes, releases mature oocytes for fertilization, and maintains pregnancy. Yak (*Bos grunniens*) is the only bovid animal that can adapt to the harsh climatic conditions on the Qinghai‐Tibetan Plateau (altitudes of over 3000 m above sea level). However, the cellular atlas is composed of oocytes and other somatic cells, and their individual molecular characteristics remain to be elucidated in the yak ovary. Here, single‐cell RNA sequencing (scRNA‐seq) was performed to delineate the molecular signature of various cell types in the yak ovarian cortex. A cellular atlas of yak ovarian cortex was constructed successfully on the basis of the differentially expressed genes (DEGs) from the distinct cell types and their functional enrichment analysis, comprising endothelial cells, nature kill cells, stromal cells, smooth muscle cells, oocytes, macrophages, epithelial cells, and granulosa cells. Meanwhile, the signature genes were determined based on their expression specificity in each cell type. A cell‐to‐cell communication network was built in light of the differentially overexpressed ligand and receptor genes from each cell type. Further, the oocytes were subdivided into four subtypes based on their individual DEGs and the functional enrichment of the DEGs. FST and TOP2A were identified as maker genes for oocytes by immunostaining in the yak ovarian cortex. The cellular atlas reveals the biological characteristics of the ovarian cortex at the cellular molecular level and provides insights into female reproductive biology via cellular communications in the yak.

**Abbreviations**
BSA: bovine serum albumin
DAPI: 4′,6‐diamidino‐2‐phenyl‐indole
DEGs: differentially expressed genes
GEM: gel beads in emulsion
GO: gene ontology
KEGG: Kyoto encyclopedia of genes and genomes
PBS: phosphate‐buffered saline
PCA: principal component analysis
PCs: principal components
RT: room temperature
scRNA‐seq: single‐cell RNA sequencing
tSNE: t‐distributed stochastic neighbor embedding
UMAP: uniform manifold approximation and projection
UMI: unique molecular identifier

## 1 INTRODUCTION

Mammalian ovary generates sex hormones to support female phenotypes, provides developmentally competent and mature oocytes, and maintains pregnancy.^1^, ^2^, ^3^, ^4^ In contrast to spermatogenesis starting from sexual maturity, oogenesis begins with fetal life. Primordial germ cells proliferate and migrate from a yolk sac to a genital ridge to become oogonia in mammalian female fetuses.^5^ The oogonia undergo several cycles of mitotic division with incomplete cytokinesis forming germ cell nests or cysts. The cysts are broken down to form primordial follicles where somatic pregranulosa cells surround oocytes.^6^ The oocytes within the primordial follicles are subsequently arrested at the diplotene stage of meiotic prophase I until the oocytes restart meiosis in puberty.^7^, ^8^ Reserve of dormant oocytes within primordial follicles of ovarian cortex determines mammalian female fecundity.^9^

Upon activation, the primordial follicles migrate toward the ovarian medulla, the inner part of the ovary.^7^, ^10^ Afterward, the ovarian follicles go through several developmental processes to reach maturity, including granulosa cell proliferation and differentiation, follicle fluid accumulation in the antrum, and theca cell generation from stromal cells.^11^, ^12^ However, the overwhelming majority of the follicles undergo a degenerative process known as atresia and follicle atresia happens at any stages of folliculogenesis and follicle development.^13^, ^14^ From two to three million primordial follicles present within a yak (*Bos grunniens*) ovary at birth, about 210 000 of which remains around puberty, whereas the rest undergoes degeneration and apoptosis before reaching ovulation. As only one dominant follicle reaches an ovulatory stage in each estrus, it is crucial for maintaining ovarian homeostasis that follicular waves are generated regularly and subsequent atretic follicles are eliminated efficiently. Ovarian tissue reconstruction occurs in each estrus cycle with the transformation of ovulatory follicles into hormone‐producing corpus luteal, followed by regression to corpus albicans.^15^, ^16^

Yak (*Bos grunniens*) is the only bovid breed that possesses strong adaptability to the harsh ecological environment with high elevation, low oxygen, severe cold, and high ultraviolet radiation on the Qinghai‐Tibetan Plateau.^17^ Domestic yak is the most important for the production and life of the local people as it provides food, shelter, fuel, and transport.^18^, ^19^ Moreover, the ovary is a vital organ for reproduction, whose normal biological function determines the reproductive efficiency of animals. The ovarian function depends on communications between various cell types within the ovary via interactions between biological molecules derived from the cells. Consequently, depicting the cellular molecular atlas and cell communications within the yak ovary can contribute to understand the precise mechanism underlying the reproductive characteristics adaptable to the plateau environment.

Single‐cell RNA‐sequencing technology has been applied to reveal cellular signatures of ovarian tissue of a few species. So far, the comprehensive ovarian cell atlases were built for humans,^20^, ^21^ monkeys,^22^, ^23^ drosophilas,^24^, ^25^, ^26^ zebrafish,^27^ seabass,^28^ and finless eel^29^ by scRNA‐seq. Specific gene signatures of oocyte and granulosa cells were identified at different developmental stages that might reflect both oocyte competency and ovarian reserve in adult human ovaries.^30^, ^31^ The cellular signature of the human fetal ovary was investigated to provide insights into the cellular network coming up during the formation of primordial follicles in fetal development.^32^ However, the molecular signatures of ovarian cells are not well known in the yak. Understanding the signatures is paramount to elucidate the reproductive adaptability to the ecological environment on the Qinghai‐Tibetan Plateau. Therefore, the cell types and associated molecular signals that regulate ovary function are expected to be uncovered in the yak.

In the present study, a comprehensive atlas composed of different cell types was constructed to remodel the cellular molecular structure of the yak ovarian cortex through scRNA‐seq. The signature genes for each cell type were identified, which might be linked to cellular functions. A cell‐to‐cell communication network between the cell types was built based on the expression levels of ligand–receptor pairs. The study delineated the molecular characterization and biological functions of the distinct cell populations within the yak ovarian cortex. The data sets are valuable for filling the gap between ovarian structure and function and deciphering the plateau adaptability of reproduction in the yak.

## 2 METHODS

### 2.1 Yak ovary collection

Healthy female yaks residing in high‐altitude regions (altitudes of over 3200 m above the sea level) were recruited at a local abattoir in Menyuan Hui Autonomous Country, Qinghai Province, China. Many adult yaks in estrus, aged from 4 to 5 years, were selected as potential experimental candidates. They were nonpregnant and free from any anatomical reproductive disorders. After slaughter, the ovaries were removed immediately from the yak carcasses. Six ovaries derived from six different yaks, devoid of any active corpus luteum, containing several antral follicles (with an outer diameter of about 2–15 mm), were selected for subsequent experiments.

### 2.2 Preparation of ovarian tissue for single‐cell RNA sequencing

A typical estrous ovary containing a dominant follicle with a diameter of 15 mm and several antral follicles with diameters under 5 mm was selected for the subsequent scRNA‐seq experiment. The fresh ovary was flushed with normal saline to remove the blood and then minced into approximate 3 mm cubed pieces with a sterile scalpel blade. A total of five ovarian cortical pieces were obtained. The pieces were washed with phosphate‐buffered saline (PBS) in a sterile culture dish. Each cubed piece was separately transferred into a cryogenic vial containing cell freezing medium (80% DMEM, 10% FBS, and 10% DMSO), resuspended adequately, and incubated at room temperature (RT) for 10 min. Then, the cryogenic vials with the tissue pieces were transferred into a gradient cooling box containing isopropanol. The gradient cooling box was buried in dry ice for 12 h, and then the cryogenic vials were put into liquid nitrogen for cryopreservation.

### 2.3 Single‐cell dissociation of ovarian tissue

The ovarian cortical samples were dissociated for single‐cell transcriptomics as previously described.^33^ Briefly, the pieces of ovarian cortex were thawed in a water bash at 37°C and washed in a cooled RPMI 1640 medium containing 0.04% bovine serum albumin (BSA) twice. The ovarian cortical pieces were further minced into approximately 0.5 mm^3^ pieces with a sterile scalpel blade in RPMI 1640 medium containing 0.04% BSA. The pieces were digested with 1 mg/ml collagenase Type II (Life Technologies, USA) in 0.25% trypsin–EDTA (Life Technologies, USA) on ice overnight. The digestion was stopped by adding 10% of fetal calf serum (Gibco). Next, the dissociated cells were collected by centrifugation at 160*g* for 3 min and incubated with advanced DMEM/F12 Glutamax (Life Technologies) containing 1% insulin‐transferrin‐selenium (Life Technologies, USA), 1% penicillin–streptomycin (Life Technologies, USA), and 27 IU/ml RNase‐free DNase I (Qiagen, Germany) at 37°C for 1 h. The ovarian cells were resuspended in DPBS containing 2% FBS and passed through a 40 μm cell strainer (Corning, USA). The cell suspension was counted and stored for subsequent experiments.

### 2.4 Single‐cell RNA sequencing

Dead cells in the cell suspension were removed by a dead cell removal kit (MACS, Milteny Biotec, Germany). The cell suspensions containing high viability cells were prepared immediately. Chromium Next GEM Single Cell 3′ Reagent Kit v3.1 (PN‐1000128, 10X Genomics, USA) was used for gel beads in emulsion (GEM) formation. The cell suspensions, gel beads, and partitioning oil were loaded into a Chromium Single Cell Controller to produce single‐cell GEMs. Then, a primer containing Illumina TruSeq Read 1, 10X Barcode, and unique molecular identifier (UMI) was applied to produce barcoded full‐length cDNA by reverse transcriptase reaction. The GEMs were broken and the pooled fractions were recovered. The first‐strand cDNAs were purified with silane magnetic beads and amplified to generate sufficient mass for library construction. Enzymatic fragmentation and size selection were used to optimize cDNA amplicon size. P5, P7, a sample index, and a TruSeq Read 2 were added to the amplicon via end repair, A‐tailing, adaptor ligation, and PCR. Subsequently, the library was sequenced on an Illumina sequencing platform (Novaseq 6000) using a 300 cycles kit (Illumina) and paired‐end readings of 150 bp were generated.

### 2.5 Primary sequencing analysis and original data generation

Raw scRNA‐seq data were converted using the Cell Ranger analysis pipeline provided by 10X Genomics (v2.2.0) and the reads were aligned to the yak genome version BosGru3.0 using the STAR aligner.^34^ Cell Ranger output in the form of a “filtered gene‐barcode” count matrix, containing the expression profiles of cells with correctly detected cellular barcodes, was used for downstream analyses.

### 2.6 Single‐cell RNA‐seq data quality control and normalization

The expression matrix of cells was handled in a workflow of R (version 4.1.3) using the R package Seurat version 4.1.0.^35^, ^36^ After the Cell Ranger filtration, cells with at least 200 genes and genes expressed in at least three cells were kept. The function “PercentageFeatureset” was applied to calculate percentages of mitochondrial, hemoglobin, and ribosomal features. The function “Vlnplot” was used to visualize the percentages of the nFeature, nCount, hemoglobin, mitochondrion, and ribosome. The scatterplots depicting correlations between the features were drawn by the function “FeatureScatter.” For filtering good quality cells, the following parameters were used to exclude cells with extreme values indicating low complexity, duplets, or apoptotic cells: total number of expressed genes per cell is less than 200 or more than 2500, and percentage of mitochondrial genes to total genes is more than 20% in each cell.

Count data per cell was normalized using the function “NormalizeData” of the R package Seurat. Briefly, the UMI counts for each gene in each cell were divided by the sum of the UMI counts of that gene across all the cells. Subsequently, the obtained values were multiplied by a scale factor (10 000) and log_e_ transformed. The top 2000 highly variable genes across single cells were selected using the variance‐stabilizing transformation selection method in the function “FindVariableFeatures”.^37^ The obtained variables were then centered to a mean of zero and scaled to a standard deviation of one with the function “ScaleData”.

### 2.7 Principal component analysis

Principal component analysis (PCA) was carried out to reduce the dimensionality of the log‐transformed gene‐barcode matrix of the top highly variable genes onto a smaller set of composite variables using the function “RunPCA”. The top 2 principal components (PCs) and their compositions were visualized by the functions “DimPlot” and “VizDimLoadings,” respectively. Heat maps of the top 2 PCs and top 15 PCs and their compositions were plotted by the “DimHeatmap” function. The “ScoreJackStraw” and “Elbowplot” functions of Seurat were used to determine how many PCs were the optimal condition for clustering, and the corresponding plots were drawn, respectively.

### 2.8 Cell clustering

After inspection of the JackStraw and Elbow plots, a shared nearest neighbor graph was constructed with the “FindNeighbors” function from the Seurat package based on the top 18 PCs. Then, cell clustering was performed using the “FindClusters” function, with a resolution parameter set to 0.6. The cell clusters were visualized by the two nonlinear dimensional reduction techniques, uniform manifold approximation and projection (UMAP), and t‐distributed stochastic neighbor embedding (tSNE). The visualizations were realized using the functions “RunUMAP” and “RuntSNE” with a perplexity parameter of 18, giving each datapoint a location on the two 2D maps.

### 2.9 Maker gene selection for each cell type

The differentially expressed genes (DEGs) were determined by performing differential expression analysis between cells of one cluster and cells of the rest clusters in the data set using the “FindAllMarkers” function of Seurat (Wilcoxon Rank Sum test). In this procedure, a log 2 FC threshold, measuring how much a feature is differentially expressed in each cell cluster, was set to 0.25. A minimum percentage of each feature expression in each cluster was set to 0.25. Upregulated DEGs with high expression percentages were considered marker genes distinguishing each cluster. A heat map and a dot plot were constructed by the functions “DoHeatmap” and “DotPlot,” respectively, to exhibit the differential expression of the marker genes in each cluster. Expression characteristics of the signature genes were displayed on UMAP plots by the “FeaturePlot” function.

### 2.10 Gene ontology and Kyoto encyclopedia of genes and genomes enrichment

Gene ontology (GO) and Kyoto encyclopedia of genes and genomes (KEGG) enrichment analyses were implemented by the Bioconductor packages “clusterProfiler” and “org.Bt.eg.db” based on the 200 most variable DEGs per cell cluster. The results of GO and KEGG were demonstrated on heat maps using the “heatmap” function from the R package “ggplot2.”

### 2.11 Cell type annotation

The cell clusters were automatically annotated using the “SingleR” package based on the high‐variable marker genes. Subsequently, the clusters were manually annotated as distinct cell types in light of the “SingleR” annotation results, CellMarker data set, previous relevant reports, and biological functions of the signature genes. The distinct annotated cell types were demonstrated in a 2D UMAP plot by the “DimPlot” function. A showcase of diagrams, including a heat map, a dot plot, nine feature plots, and nine violin plots, was produced by the “DoHeatmap”, “DotPlot”, “FeaturePlot”, and “VlnPlot” functions to display expression specificity of the representative genes in each cell type.

### 2.12 Construction of developmental trajectory

To understand the transcriptional dynamics that occurred in cells, pseudotime trajectory analysis was performed to predict continuous cell states in the yak ovary. The pseudotime algorithm remodels molecular state transitions of a continuous process by quantifying the gradual divergence of single‐cell transcriptomes individually. The R package monocle was implemented to order cells in their trajectory along pseudotime.^38^ A monocle object was constructed by the function “newCellDataSet” of the package “monocle” with lowerDetectionLimit = 0.5. The pseudotime trajectories of the distinct cell types were a plot by the function “plot_cell_trajectory.” Heatmaps were generated with the “plot_pseudotime_heatmap” function for the highly variable genes and signature genes along the pseudotime.

### 2.13 Cell communication analysis

Cell communications between the different cell types were systematically analyzed with the “CellPhoneDB” Python package. Ligand–receptor interactions were inferred for each pair of cell types, and interplays between the cell types were explored by pairwise comparisons. Only the situation that receptors and ligands expressed in more than 10% of the tested cell types were considered to be an existence of interaction between the cell types. The general links and strengths of the interplays between the cell types were visualized using the “circlize” and “heatmap” R package. The top significant ligand–receptor interactions via which the oocytes communicate with the other cell types are exhibited in a bubble plot generated using the “ggplot2” R package.

### 2.14 Hematoxylin and eosin staining

After removal, six ovaries were washed with cold normal saline and further cut into approximate 5 mm cubed pieces with a sterile scalpel blade. The cubed pieces of ovarian tissue were immediately rinsed with PBS and fixed in 4% methanol‐free paraformaldehyde. The ovarian tissue pieces were dehydrated by successive incubations in 70%, 80%, 90%, and 100% ethanol and xylene. The dehydrated tissue pieces were placed into plastic cassettes, where they were embedded in two changes of paraffin at 65°C. The ovarian tissue pieces were left at RT until the paraffin solidified. The paraffin‐embedded tissue pieces were cut into 4 μm sections on an RM2065 microtome (Leica Instruments GmbH, Germany), floated in a warm water bath, mounted onto slides, and transferred to a 65°C oven for 16 h. The sections were deparaffinized in three successive xylene baths for 5 min each and rehydrated through a descending series of alcohol rinses (100%, 90%, 70%, 50%, and 25% ethanol) for 5 min each. Subsequently, the sections were stained with hematoxylin for 15 min, followed by a rinse with running tap water for 2 min. The deparaffinized sections were incubated with 0.3% acid alcohol for 10 s, rinsed with running tap water for 2 min, and transferred into 0.6% ammonium hydroxide aqueous solution until a blue color appeared, followed by another rinse with running tap water for 2 min. Then, the sections were placed in 85% alcohol for 3 min, stained with eosin for 2 min, and rinsed under running tap water for 5 s. The sections were processed through the same dehydration series as indicated above. Finally, the sections of ovarian tissue were cleared in xylene, sealed with a neutral gum seal, and imaged using a microscope approximately 24 h after staining.

### 2.15 Immunofluorescence validation

The ovarian tissue samples (3–5 mm diameter) from three yak were fixed overnight in 4% paraformaldehyde at 4°C, transferred to 70% ethanol, and embedded in paraffin using a Shandon Excelsior tissue processor (Thermo Scientific, UK). The paraffin‐embedded tissue blocks were sectioned (4 μm thickness) using a microtome onto StarFrost slides. For immunofluorescence staining, the tissue sections were deparaffinized in xylene twice, rehydrated through a series of ethanol (100%, 90%, 80%, and 70%), and ended with distilled water at RT. Antigen retrieval was performed in 0.01 M sodium citrate buffer (pH 6.0) at 98°C for 20 min. After cooling down, the sections were rinsed three times with PBS and blocked in a blocking buffer (1% BSA, 0.05% Tween‐20 in PBS) at RT for 1 h. Subsequently, the sections were incubated with the primary antibodies rabbit anti‐FST (1:200, bs‐21618R, Bioss, China) and rabbit anti‐TOP2A (1:200, bs‐1920R, Bioss, China) at RT overnight, followed by incubation with the goat anti‐rabbit IgG secondary antibody (1:500, A16118, ThermoFisher, USA) in blocking buffer at RT for 1 h. Cell nuclei were stained with 4′,6‐diamidino‐2‐phenyl‐indole (DAPI, Life Technologies, USA), and sections were mounted using ProLong Gold (Life Technologies, USA). The stained tissue sections were mounted in fluorescent mounting media (Dako Agilent, USA). The sections were scanned with Pannoramic 250 Flash III digital scanner (3DHISTECH Ltd., Hungary), and the representative areas were selected for imaging using “Pannoramic Viewer” software. The immunofluorescence detection was performed in technical triplicates.

## 3 RESULTS

### 3.1 Data quality control and principal component analysis

**FIGURE 1:**
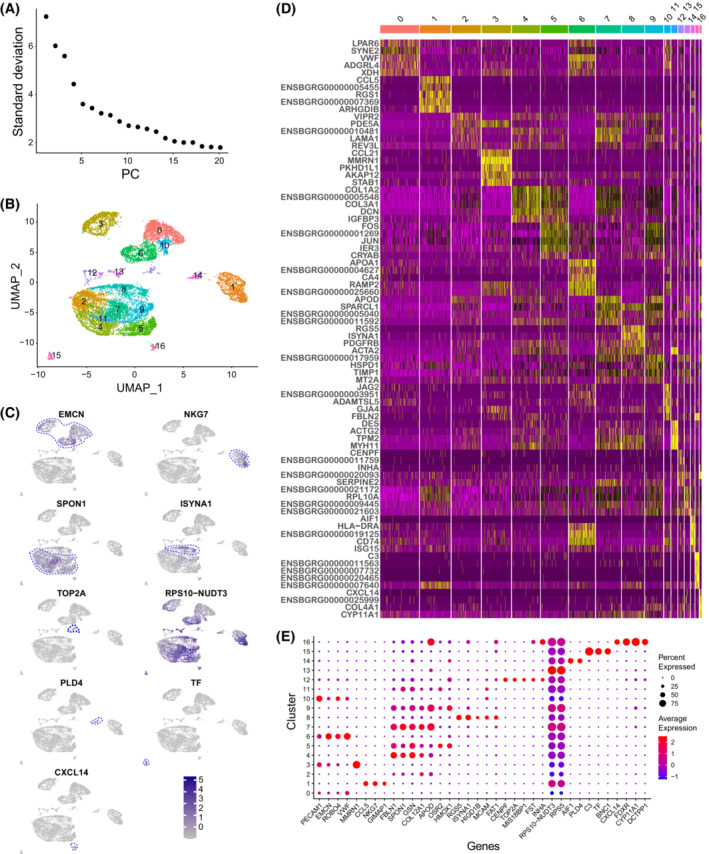
Clustering analysis on various cells in yak ovarian cortex. (A) The elbow plot shows standard deviations of the top 20 principle components (PCs) in principle component analysis (PCA). (B) Uniform manifold approximation and projection (UMAP) scatterplot visualizing cell clusters. Each point corresponding to a single cell is color‐coded according to its cluster membership. (C) Feature plots demonstrating expression specificity of the signature genes across all the ovarian cortical cells. The expression level of each gene from none to high is indicated by a color gradient from light gray to dark blue. Blue dashed lines give boundaries of the main clusters of interest. (D) Heat map exhibiting distinct expression patterning of the five signature genes for each cluster across the entire data set. The expression level of each gene from low to high is indicated by a color gradient from purple to yellow. (E) The dot plot shows distinct expression patterning of the selected signature genes for each cluster. The expression level of each gene from low to high is indicated by a color gradient from blue to red. The percentage of cells expressing a specific gene is indicated by the size of a dot.

The percentages of the nFeature, nCount, hemoglobin, mitochondrion, and ribosome (Figure S1) were successfully applied to quality control. After applying the quality control filters, a total of 11 853 single cells expressing 17 682 genes were retained for downstream analyses. The data set was normalized and scaled subsequently. PCA was performed on the scaled data set. The number of PCs was chosen through visualization in the elbow plot (Figure 1A), and the top 18 PCs were used to perform downstream analyses.

### 3.2 Cell clustering and marker gene selecting

A K‐nearest neighbor graph was constructed based on the Euclidean distance in PCA space. The top 18 PCs were chosen for cell clustering. A modularity optimization technique (Louvain algorithm) was employed to cluster cells, and the most optimal resolution parameter of 0.6 was used after optimizing the parameter ranging from 0.4 to 1.2. Then, cell clusters were visualized through the nonlinear dimensionality reduction algorithm UMAP in a two‐dimensional plot. The ovarian cortical cells were clustered into 17 cell clusters (Figure 1B). The top 30 DEGs from each cluster were filtered by their adjusted *p* values (Wilcoxon rank sum test) (Data S1). The top 5 variable features of each cluster are shown in a heatmap (Figure 1D), indicating their expression specificity among the 17 clusters. The expression distributions of the representative DEGs, including EMCN, NKG7, SPON1, ISYNA1, TOP2A, RPS10‐NUDT3, PLD4, TF, and CXCL14, are demonstrated in nine UMAP plots (Figure 1C). The expression levels and percentages of the genes of interest across the different clusters are visualized in a dot matrix plot (Figure 1E). The above analysis of gene expression specificity indicates that each cell cluster possesses many specifically expressed genes which may be considered marker genes.

### 3.3 Cell type identification

**FIGURE 2:**
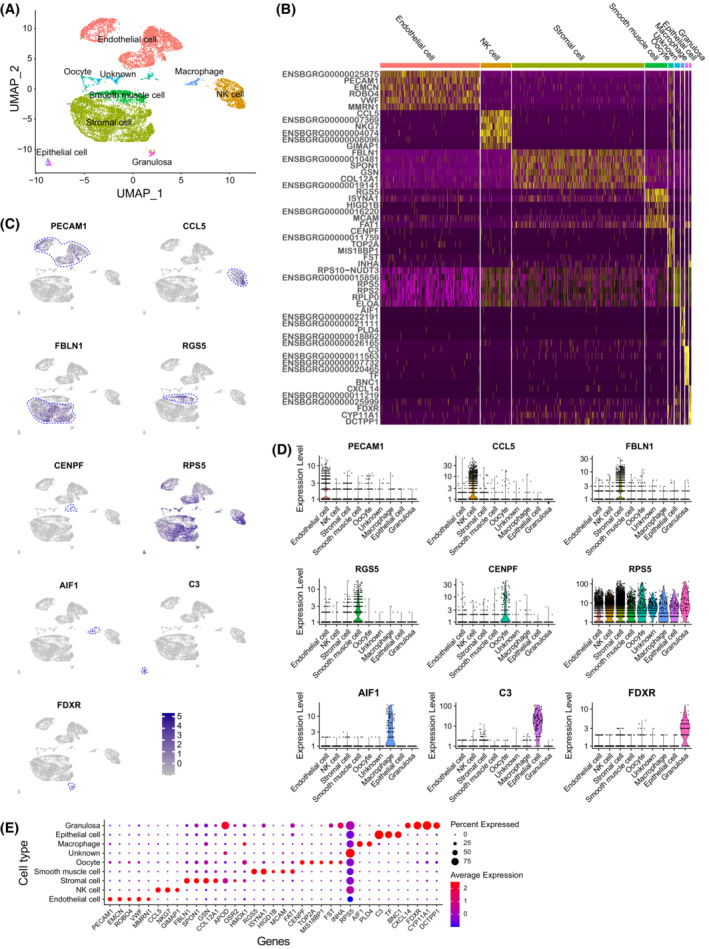
Determination of various cells in yak ovarian cortex. (A) Uniform manifold approximation and projection (UMAP) scatterplot visualizing cell types. Each point corresponding to a single cell is color‐coded according to its cell type membership. (B) Heat map exhibiting distinct expression patterning of the six signature genes for each cell type across the entire data set. The expression level of each gene from low to high is indicated by a color gradient from purple to yellow. (C) Feature plots demonstrating expression specificity of the signature genes across all the ovarian cortical cells. Expression level of each gene from none to high is indicated by a color gradient from light gray to dark blue. Blue dashed lines give boundaries of the main cell types of interest. (D) Violin plots visualize the expression specificity of the signature genes for each cell type. Expression values of the signature genes were scaled by log‐normalization. The vertical coordinate displays the expression scores of the signature genes. (E) The dot plot shows distinct expression patterning of the selected signature genes for each cell type. The expression level of each gene from low to high is indicated by a color gradient from blue to red. The percentage of cells expressing a specific gene is indicated by the size of a dot.

Although the “SingleR” package provides an automatic method to annotate scRNA data, it seemed not suitable to be employed in yak cell type annotation because the annotation for the yak ovarian cells could not reflect the truth (Figure S2). In view of this, manual identification was carried out based on the functions of the signature genes in each cluster. Then, the cell clusters were annotated successfully into nine cell types including oocytes (one cluster, 1.84%), granulosa cells (one cluster, 0.68%), stromal cells (six clusters, 43.46%), endothelial cells (four clusters, 32.70%), smooth muscle cells (one cluster, 7.28%), NK cells (one cluster, 9.99%), macrophages (one cluster, 1.17%), epithelial cells (one cluster, 1.09%), and unknown cells (one cluster, 1.79%) (Figure 2A). To further verify the cell types in the clusters, the expression specificity of six representative DEGs per cell type is demonstrated in a heat map (Figure 2B). Most of the DEGs express specifically in their own cell types. The expression levels of the nine most presentative cell type‐specific genes, including PECAM1, CCL5, FBLN1, RGS5, CENPF, RPS5, AIF1, C3, and FDXR, overlay on UMAP plots (Figure 2C). Each of them possesses a higher and more specific expression in its individual cell type except RPS5 highly expressed in even every cell type. In line with the heat map and feature plots, the distinct cell types are exclusively identified by their signature genes except for unknown cell clusters in violin plots (Figure 2D). Expression scores and percentages of the specifically expressed genes across the different cell types are visualized in a dot matrix (Figure 2E). The inspection of gene expression specificity indicates that the cell type‐specific genes CENPF, FDXR, FBLN1, PECAM1, RGS5, CCL5, AIF1, and C3 can be considered marker genes for oocytes, granulosa cells, stromal cells, endothelial cells, smooth muscle cells, NK cells, macrophages, epithelial cells, respectively.

### 3.4 Gene ontology and Kyoto encyclopedia of genes and genomes enrichment for all the cell types

**FIGURE 3:**
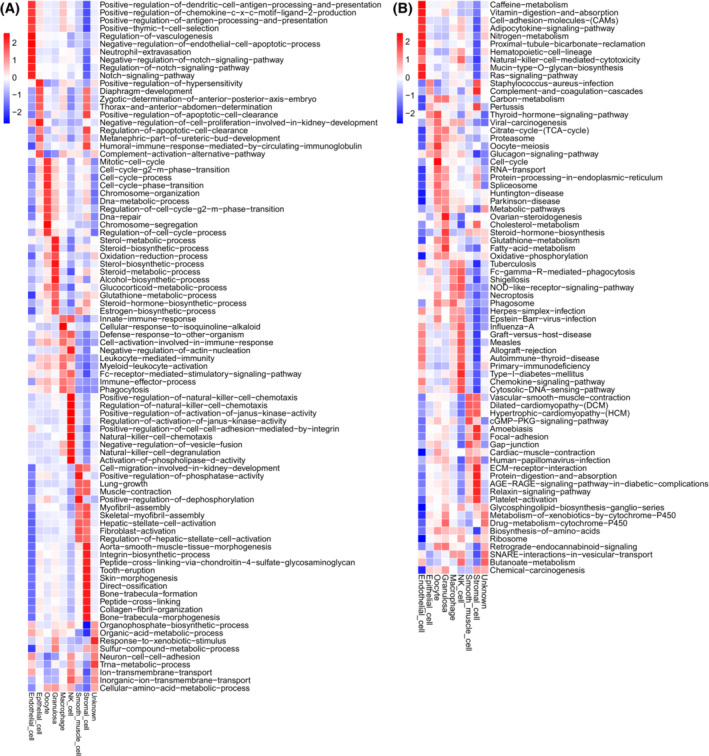
Gene ontology and Kyoto encyclopedia of genes and genomes enrichment for the cell types in yak ovarian cortex. (A) Heat map exhibiting enriched biological process (BP) terms from gene ontology (GO) analysis. The enrichment score of each BP term from low to high is indicated by a color gradient from blue to red. (B) Heat map exhibiting enriched pathway terms from Kyoto encyclopedia of genes and genomes (KEGG) analysis. The enrichment score of each pathway term from low to high is indicated by a color gradient from blue to red.

Following the GO enrichment analysis, a heat map was produced based on the 10 biological processes with the highest enrichment FDRs per cell type, distinguishing the biological functions of the nine major cell types (Figure 3A). The DEGs highly expressed in the endothelial cells mainly participate in vasculogenesis, endothelial cell apoptosis, notch signaling pathways, etc. The oocytes tend to be involved in cell cycle transition, DNA repair, and chromosome segregation processes. The granulosa cells are involved in sterol, steroid, glucocorticoid, estrogen metabolism, and oxidation–reduction processes. In brief, the GO enrichment indicates that the biological processes in which each cell participates coincide with its known biological functions. However, the unknown cells cannot be identified because the pathways that they take part in are also enriched in the other cell types. In line with the GO enrichment, the KEGG enrichment demonstrates that the oocytes play a role in oocyte meiosis and cell cycle pathways, and the granulosa cells participate in steroidogenesis, cholesterol metabolism, steroid hormone biosynthesis, and oxidative phosphorylation pathway (Figure 3B).

### 3.5 Pseudotime expression patterns for representative genes in all the cell types

**FIGURE 4:**
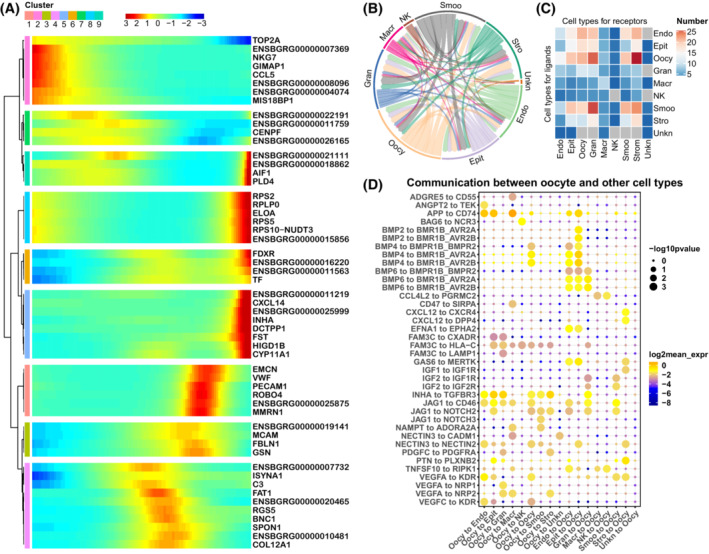
Cell‐to‐cell communication in yak ovarian cortex. (A) Pseudotime‐ordered heat map demonstrating of pseudotime order of the selected marker genes for all the cell types. The expression level of each gene from low to high is indicated by a color gradient from blue to red. (B) Network plot demonstrating the general information of cell‐to‐cell communications depending on significant ligand–receptor pairing interactions and their direction as autocrine or paracrine signals. (C) Heat map showing the strength of interactions between cell types based on the amounts of ligands or receptors. The number of ligands or receptors from small to large is indicated by a color gradient from blue to red. (D) Putative communications between oocytes and the other cell types through ligand–receptor interactions. Endo, endothelial cell; Epit, epithelial cell; Gran, granulosa cell; Macr, macrophage; NK, natural killer cell; Oocy, oocyte; Smoo, smooth muscle cell; Stro, stromal cell; Unkn, unknown cell.

The pseudotime of each cell type is determined by its mapped position along the principal trajectory (Figure S3). A heat map of the representative genes shows their dynamic expression along the pseudotime, indicating the temporal and progressive dynamics of the genes (Figure 4A). The high expression of the genes implies that they were executing their individual biological functions at the individual stages of the pseudotime period. As shown in the heat map, the expression of the marker gene CCL5 for NK cells starts at a high level at the initial stage of the pseudotime period and goes down gradually at the subsequent stages. The abundance of the marker genes PECAM1, FBLN1, RGS5, and C3 are upregulated at the middle stage of the pseudotime period while those of AIF1 and FDXR are raised dramatically at the late stage. Different from the above genes, the expression level of the marker gene CENPF for oocytes descends progressively during the whole pseudotime period. Oogenesis undergoes three distinct developmental stages: meiotic initiation in the fetus, follicle formation in the perinatal period, and oocyte growth and maturation in the adult. The oocytes within the yak ovarian cortex stay in a quiescent state or a growth state. CENPF is involved in the regulation of DNA synthesis and hence cell cycle progression. The result implies that CENPF may participate in regulating the DNA synthesis and cell cycle of the yak oocytes, and its function gradually weakens during the pseudotime period.

### 3.6 Cell communication analysis for cell types

The receptor–ligand interactions between annotated cell types were inferred based on the high expression of a ligand by one cell type and a corresponding receptor by another cell type (Data S2). A cell‐to‐cell communication network was built on the basis of the numbers of the ligands and their receptors (Figure 4B, Data S3). As shown in the network, the oocytes principally regulate the stromal cells, granulosa cells, and smooth muscle cells and are mainly controlled by the endothelial cells, smooth muscle cells, epithelial cells, and themselves. The closeness of regulatory relationships between cell types is exhibited in a heat map based on the amounts of the ligands and their receptors (Figure 4C). The large number of ligands are produced by the oocytes corresponding to the receptors produced by the stromal cells and granulosa cells, indicating that the oocytes mainly regulate the two cell types. There are relatively larger numbers of the receptors produced by the oocytes corresponding to the ligands by the endothelial cells and themselves, suggesting that the oocytes are primarily controlled by the endothelial cells and themselves. The top significant interactions between the receptors and ligands are demonstrated in a bubble plot, indicating the communications between the oocytes with other cell types (Figure 4D). The oocytes regulate the other cell types mainly via their ligands FAM3, INHA, and JAG1 recognizing their corresponding receptors. Oppositely, the oocytes are controlled by the endothelial cells, epithelial cells, and granulosa cells principally through the BMP family. Moreover, the granulosa cells control the oocytes via the ligands IGF2, INHA, and JAG1 recognizing their corresponding receptors.

### 3.7 Potential functional heterogeneity of oocytes

**FIGURE 5:**
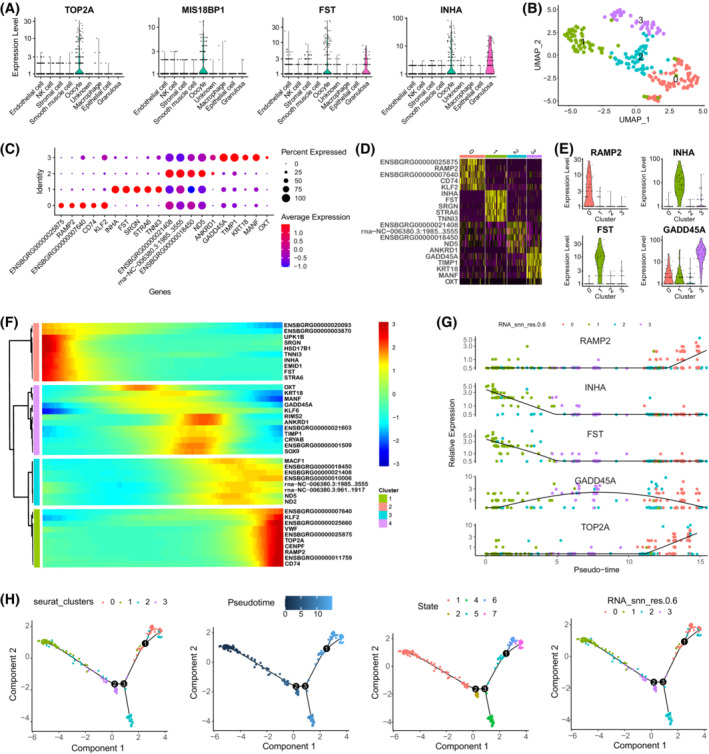
Clustering and pseudotime analyses for yak oocyte. (A) Violin plots visualize the expression specificity of the signature genes for each oocyte subtype. Expression values of the signature genes were scaled by log‐normalization. The vertical coordinate displays the expression scores of the signature genes. (B) Uniform manifold approximation and projection (UMAP) scatterplot visualizing oocyte subtypes. Each point corresponding to a single cell is color‐coded according to its subtype membership. (C) Dot plot showing distinct expression patterning of the selected signature genes for each oocyte subtype. The expression level of each gene from low to high is indicated by a color gradient from blue to red. The percentage of cells expressing a specific gene is indicated by the size of a dot. (D) Heat map exhibiting distinct expression patterning of the five signature genes for each oocyte subtype. The expression level of each gene from low to high is indicated by a color gradient from purple to yellow. (E) Violin plots visualize the expression specificity of the signature genes for each oocyte subtype. Expression values of the signature genes were scaled by log‐normalization. The vertical coordinate displays expression scores of the signature genes. (F) Pseudotime‐ordered heat map demonstrating of pseudotime order of the selected marker genes for oocyte subtypes. The expression level of each gene from low to high is indicated by a color gradient from blue to red. (G) Expression plots demonstrating expression trends of the signature genes in oocyte subtypes arranged along pseudotime. (H) Scatterplot showing the differential trajectories of four oocyte subtypes with pseudotime scale by Monocle.

Around 1.84% of the ovarian cortical cells were annotated as oocytes. Besides CENPF, the signature genes TOP2A, MIS18BP1, FST, and INHA are more highly expressed in the oocytes than those in the other cell types (Figures 2D, 5A). In the heterogeneity analysis, the oocytes were subdivided into four different subtypes (Figure 5B). The DEGs for each subtype were filtered out. The expression differences of the top 5 DEGs for each subtype are shown in a dot plot and a heat map plot (Figure 5C,D). As shown in the plot, clusters 0, 1, and 3 specifically express RAMP2, INHA, and GADD45A, respectively, whereas cluster 2 lacks specific signature genes (Figure 5E).

To study the transcriptomic paths that the oocytes would take during their differentiation process, the developmental trajectory of the oocytes was constructed. The dynamic expression of the potential marker genes is demonstrated along the pseudotime in a heat map (Figure 5F). As shown in the heat map, the expression levels of INHA and FST are upregulated at the early stage and are downregulated afterward. GADD45A is just relatively upregulated in the middle period of the pseudotime. The expression of RAMP2 and TOP2A maintain medium intensities at the early and middle stages and increase significantly in the late period. Similar trends of the presentative genes can be found in the gene expression patterns along the pseudotime axis (Figure 5G). The differential trajectories of the four oocyte subtypes are demonstrated with a pseudotime scale in a scatterplot (Figure 5H). The DEGs of the oocyte subtypes were further enriched in the GO terms and KEGG pathways (Figure S4).

### 3.8 Validation of the marker genes of the oocytes

**FIGURE 6:**
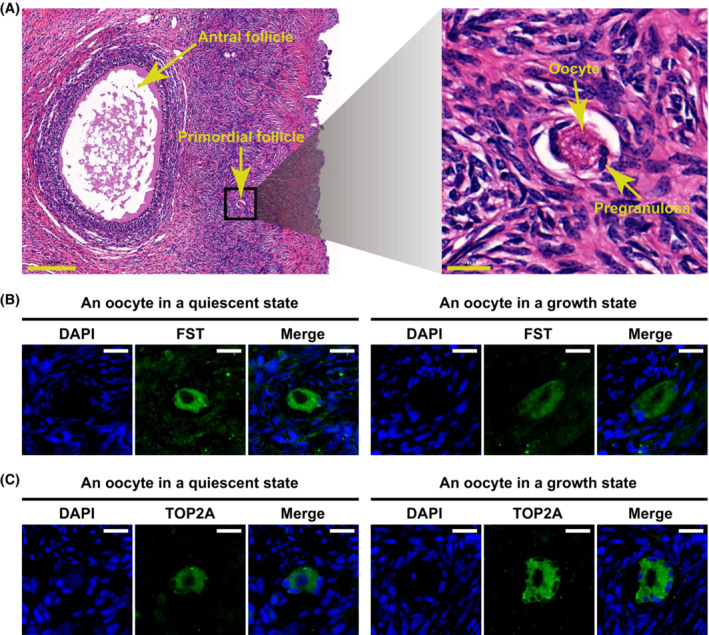
Hematoxylin and eosin (HE)‐stained section and immunofluorescence detection for yak ovarian cortex. (A) HE‐stained section exhibiting morphological and histological characteristics of yak ovarian cortex. The scale bars at left and right are 200 μm and 20 μm in length, respectively. (B and C) Sections detected by immunofluorescence demonstrating validation of the two markers FST (B) and TOP2A (C) for yak oocytes in quiescent and growth state. 4′,6‐diamidino‐2‐phenyl‐indole (DAPI) was used as a nuclear counterstain. The scale bars are 20 μm in length.

As the histological sections stained with hematoxylin and eosin exhibited, the oocytes are topographically located in the primordial follicles of the ovarian cortex (Figure 6A). Each oocyte is surrounded by a few fusiform pregranulosa cells in a preantral follicle. The reliable immunofluorescence technique was applied to determine whether the scRNA‐seq accurately screened the specifically expressed genes for the oocytes. As shown in the immunofluorescence sections, the proteins FST and TOP2A from oocytes can be successfully identified in the primordial follicles. In detail, the expression of the two proteins is located in the oocyte cytoplasm (Figure 6B,C). The expression level of FST is higher in the oocyte of quiescent state than that in the oocyte of the growth state. On the contrary, TOP2A keeps a relatively low intensity in the oocyte of quiescent state and raises its expression evidently in the oocyte of growth state. The results of immunofluorescence corroborate with the expression trends of the two genes in scRNA‐seq analysis.

## 4 DISCUSSION

Remodeling of the mammalian ovary is crucial for understanding folliculogenesis, ovulation, and corpus luteum formation processes. Different cell types were identified in growing and regressing follicles of adult human ovaries, including granulosa cells, theca/stromal cells, endothelial cells, smooth muscle cells, and immune cells.^21^ In the human ovarian cortex, six main cell types, containing oocyte, granulosa cell, stromal cell, endothelial cell, perivascular cell, and immune cell, were discovered.^20^ Similarly, the monkey ovary is comprised of oocytes, granulosa cells, stromal cells, endothelial cells, smooth muscle cells, NK cells, and macrophages.^22^, ^23^ Furthermore, besides the above cell types, epithelial cells were identified in embryonic and neonatal mice ovaries.^39^, ^40^ In the present study, the cell classification results are consistent with the known ovarian cell types present in the literature, including oocytes, granulosa cells, stromal cells, endothelial cells, smooth muscle cells, NK cells, macrophages, and epithelial cells (Figure 2A). These results suggest that cell types of the ovary are relatively conservative among various mammals. Similar to the above animals, theca cells cannot be distinguished from stromal cells due to a lack of marker genes with high discriminability in the yak.

Consistent with some reports^41^, ^42^, ^43^ and clinical studies^44^ that contradicted the existence of oogonial stem cells within the ovary, the present study did not find any signature genes of the oogonial stem cells, suggesting none of that cell type remained in the yak ovarian cortex. However, the possibility cannot be excluded that the oogonial stem cells were so rare that the analysis of the yak ovarian cells might not be sufficient to capture them. Furthermore, oogonial stem cells were presumably sensitive to the tissue treatment or cell dissociation and hence got lost during the sample processing.

Most oocytes stem from the ovarian cortex where a large number of primordial follicles constitute an ovarian reserve.^45^ After obtaining single‐cell suspension, the suspended cells usually undergo a filtering step with a 40‐μm cell strainer to eliminate larger particles in a canonical protocol of scRNA‐seq. Although the single‐cell suspension of yak ovarian cortex was also filtered prior to the subsequent protocols, most of the oocytes might have been captured from the suspension as almost all of them were under 35 μm in diameter.

DDX4 located in the cytoplasm of oocytes maintains a high expression level during germ cell development,^43^, ^46^ especially oocytes within preantral follicles.^20^ Besides DDX4, expression of GDF9, FIGLA, and ZP3 were shown to be highly specific to oocytes of humans, monkeys, and mice.^20^, ^23^, ^39^, ^40^ However, specific expression of these markers was not found in the oocytes of the ovarian cortex in the yak. Instead, CENPF, TOP2A, MIS18BP1, FAST, and INHA were characteristically expressed in the yak oocytes (Figures 2D and 5A), which can be considered marker genes for the yak oocytes in primordial follicles. Moreover, FST and TOP2A were only localized via immunofluorescence to the cytoplasm of oocytes but not to somatic cells including pregranulosa cells in the ovarian cortex (Figure 6B,C). In primates, stage‐specific regulatory networks revealed a core hub of genes that regulated cell type‐specific markers at each stage of oocyte development.^23^ In the present study, the yak oocytes were classified into four subtypes based on the well‐characterized marker genes in their corresponding clusters (Figure 5B). The unique transcriptional uniqueness of the four oocyte subtypes was displayed based on their unique molecular signatures (Figure 5D,E). These results indicated that oocytes are kept in various states within primordial follicles of the yak ovarian cortex.

A mammalian ovarian follicle consists of an innermost oocyte, surrounding granulosa cells, and thecal cells of outer layers. Gonadotropin‐sensitive granulosa cells in growing follicles are responsible for steroidogenesis which plays a crucial role in ovarian function.^47^ CYP19A1 can convert androgens to estrogens in granulosa cells.^48^ However, there was no high expression of CYP19A1 but CYP11A1 was found in the yak granulosa cells (Figure 2E). The result implies that the conversion of androgens to estrogens is not necessary for granulosa cells in the ovary during estrus yak. Furthermore, conversion of cholesterol to pregnenolone may be required for the granulosa cells which is catalyzed in a mitochondrial inner membrane by cytochrome P450 proteins encoded by CYP11A1. Conversion is the first rate‐limiting step in the synthesis of steroid hormones.

Primordial follicles that envelop immature oocytes in meiotic arrest in the ovarian cortex are independent of gonadotrophins and steroidogenically inactive.^20^ In the mammalian ovary, LH targets theca cells of antral follicles to stimulate steroidogenesis, elevating the gene levels of STAR, CYP11A1, HSD3B1, HSD3B2, and CYP17A1 through PKA signaling and synthesizing androgens and progesterone.^49^ The expression of STAR and CYP17A1 enables theca cells to synthesize androgens at the secondary follicle stage.^50^ Cells expressing STAR and CYP17A1 offer a possibility of the presence of theca cells.^51^ However, it is difficult to distinguish theca cells from stromal cells resulting from the close gene profiles between stromal cells and theca cells, even in large secondary follicles with a visible theca cell layer.^21^ Cell‐specific expression of the two signature genes were not found in any yak cell cluster yet. Meanwhile, none of the other specific genes distinguishing theca cells from the stromal cells was discovered in any stomal cell cluster, indicating there is a close relationship between theca cells and general stromal cells in the yak ovarian cortex. The factors transforming stromal cells to theca cells need to be identified in the yak ovary in further research.

To ensure follicle maturation and ovary reconstruction, new blood vessels composed of endothelial and perivascular cells are intensively forming in the ovary.^52^, ^53^ Endothelial cells in the ovary could be identified based on specific expression of a marker VWF in primates.^20^, ^23^ But the signature gene is not expressed specifically in ovaries of other species, such as a mouse,^40^, ^54^ sole,^55^ seabass,^28^ and zebrafish.^27^ Nearly, one‐third of the cortical cells was identified as endothelial cells of which only a portion expressed VWF specifically (Figure 1E), suggesting the heterogeneity of the endothelial cells in the cortex of the yak ovary. The whole group of endothelial cells was identified based on the expression of strong endothelial marker PECAM1 (Figure 1E), which is associated with lymph and blood vascular system in humans.^21^ Similarly, monkey endothelial cells possess the specific gene PECAM1 in their ovary.^22^ These studies related to the marker genes for endothelial cells further confirm the reliability of the identification of endothelial cells in the present study.

Perivascular cells include pericytes and smooth muscle cells originating from a common progenitor, one or both of which can be found up to the type of blood vessel (fine capillary or big artery).^56^ Pericytes are generally solitary cells partially covering small‐diameter blood vessels (arterioles, capillaries, and venules), whereas vascular smooth muscle cells are constituent cells of large blood vessels where they form a continuous coating.^57^ The perivascular cells regenerate in each estrus cycle in mammalian ovaries. Around 7.28% of the cells are annotated as smooth muscle cells specifically expressing RGS5 and ISYNA1 in the yak ovarian cortex (Figure 2). The expression of the marker gene RGS5 is also restricted to smooth muscle cells of ovaries in human.^20^, ^21^ However, RGS5 is not specifically expressed in smooth muscle cells of ovaries in other species.^23^, ^27^, ^28^, ^54^, ^55^ The results suggest that there is smooth muscle cell heterogeneity across species and similar characteristics of smooth muscle cells in yak to those in human.

The biological processes resulting in ovulation include ovary reconstruction, chemotaxis, microcirculatory vasomotion, and oocyte–cumulus complex forming, which are regulated by a cytokine‐mediated inflammatory response orchestrated by several immune cells, such as lymphocytes, granulocytes, and macrophages.^58^ The immune cells have been reported to participate in folliculogenesis and ovulation in the ovary reconstruction process during estrus.^59^, ^60^ Of note, the transcriptional signature of immune cells was distinguished from those of the other cell types through their distinct transcriptome patterns in yak (Figure 2). The immune cells include natural killer T cells and macrophages, composing 9.99% and 1.17% of the cortical cell population, respectively. The low ratios of immune cells are consistent with a previous study in which the ovarian cortex is primarily composed of stromal cells and endothelial cells and the ovarian medulla mainly consists of smooth muscle cells and immune cells.^23^

Complement system members including C1R, C1S, C7, and SERPING1 are upregulated markedly in theca cells from atretic follicles compared with those from growing follicles in bovine.^61^ In that research, inflammatory response rather than cell death characterized the atretic theca cells. The activation of the complement system can also take place in the human ovary.^21^ The complement system may be crucial for the physiological homeostasis of ovulation and follicle reconstruction. Although theca cells were not identified in the yak ovarian cortex, the two complement members C3 and SERPING1 expressed specifically in the yak epithelial cells suggesting that the function of epithelial cells in the yak ovarian cortex overlaps with that of theca cells in ovaries from other mammals. In addition, granulosa cells seem to execute some functions of innate immune cells in ovulatory follicles.^62^ Nevertheless, the immune mechanisms of follicle reconstruction and regressing remain to be elucidated in the yak.

Although the present study constructed a general cell atlas of the ovarian cortex in yak estrus, there are still some limitations that should be taken into account. Only one ovary was selected as the experimental sample might have caused inaccurate conclusions if the sample was not representative of the yak ovaries in estrus. Moreover, although the ovary was collected in the yak estrus, the state of the ovary might have altered subtly during the estrus. Studies with a larger sample size are expected to confirm the findings of the present study. Furthermore, the marker genes found in the present study remain to be identified by reliable experimental methods, particularly of the novel markers. For more reliable results, an in‐depth study is further required to more accurately understand the functions of the marker genes in the yak ovarian cortex. In addition, the cell type proportions derived from the present study may not necessarily reflect the true cell type proportions in yak ovarian tissue, as sampling sites, tissue handling, and dissociation methods potentially affect the cell type ratios to some extent.

## 5 CONCLUSION

In summary, the adult yak ovarian cortex consists of nine cell types, including endothelial cell, nature kill cell, stromal cell, smooth muscle cell, oocyte, macrophage, epithelial cell, granulosa cell, and unknown cell. Several previously unreported specific markers were identified for the cell types within the yak ovarian cortex. The genes FST and TOP2A can be considered molecular signatures for oocytes within primordial follicles in the yak. The network of cell‐to‐cell communications was constructed that demonstrates the interplay between the identified cell types. The results arising from the present study lay a foundation for quantitative assessment of oocyte quality and reproductive age, offer a potential molecular mechanism underlying ovary reconstruction during estrus, and provide an insight into the plateau adaptability of female reproduction in the yak.

## AUTHOR CONTRIBUTIONS

Xian Guo and Jie Pei conceived and designed the research. Jie Pei, Lin Xiong, Shaoke Guo, Xingdong Wang, Pengjia Bao, and Xiaoyun Wu collected the biological materials. Jie Pei, Lin Xiong, Shaoke Guo, and Xingdong Wang carried out the biochemical and molecular experiments. Jie Pei performed the data analysis and prepared the figures. Xian Guo performed supervision. Jie Pei wrote the original draft. Xian Guo, Jie Pei, and Ping Yan revised the manuscript. All the authors approved the final version of the manuscript.

## FUNDING INFORMATION

This work was supported by the National Natural Science Foundation of China (Grant/Award Number: 32272852), the China Agriculture Research System of MOF and MARA (Grant/Award Number: CARS‐37), and the Innovation Project of Chinese Academy of Agricultural Sciences (Grant/Award Number: 25‐LZIHPS‐01).

## DISCLOSURES

The authors declare that they have no conflict of interest.

## ETHICAL STANDARDS

All the yaks were handled in strict accordance with good animal practices by following the Animal Ethics Procedures and Guidelines of the People's Republic of China. The present study was approved by the Animal Administration and Ethics Committee of Lanzhou Institute of Husbandry and Pharmaceutical Sciences of Chinese Academy of Agricultural Sciences (Permit No. SYXK‐2020‐0026).

## Supporting information


Figure S1.

Figure S2.

Figure S3.

Figure S4.

Data S1:

Data S2:

Data S3:

## Data Availability

Raw single‐cell RNA‐sequencing data can be accessed from the NCBI Gene Expression Omnibus database (BioProject ID: PRJNA862486). Single‐cell expression data can be explored online at (https://www.ncbi.nlm.nih.gov/geo/query/acc.cgi?acc=GSE209794).
