# Supplementary material for: A single‐cell transcriptomic atlas characterizes cell types and their molecular features in yak ovarian cortex
Source: FASEB J. 2022 Dec 17;37(1):e22718. doi: 10.1096/fj.202201176RR (PMC13281852; doi:10.1096/fj.202201176RR)
Supplement: Supplementary file 2 — Figure S2. [file FSB2-37-e22718-s003.pdf]

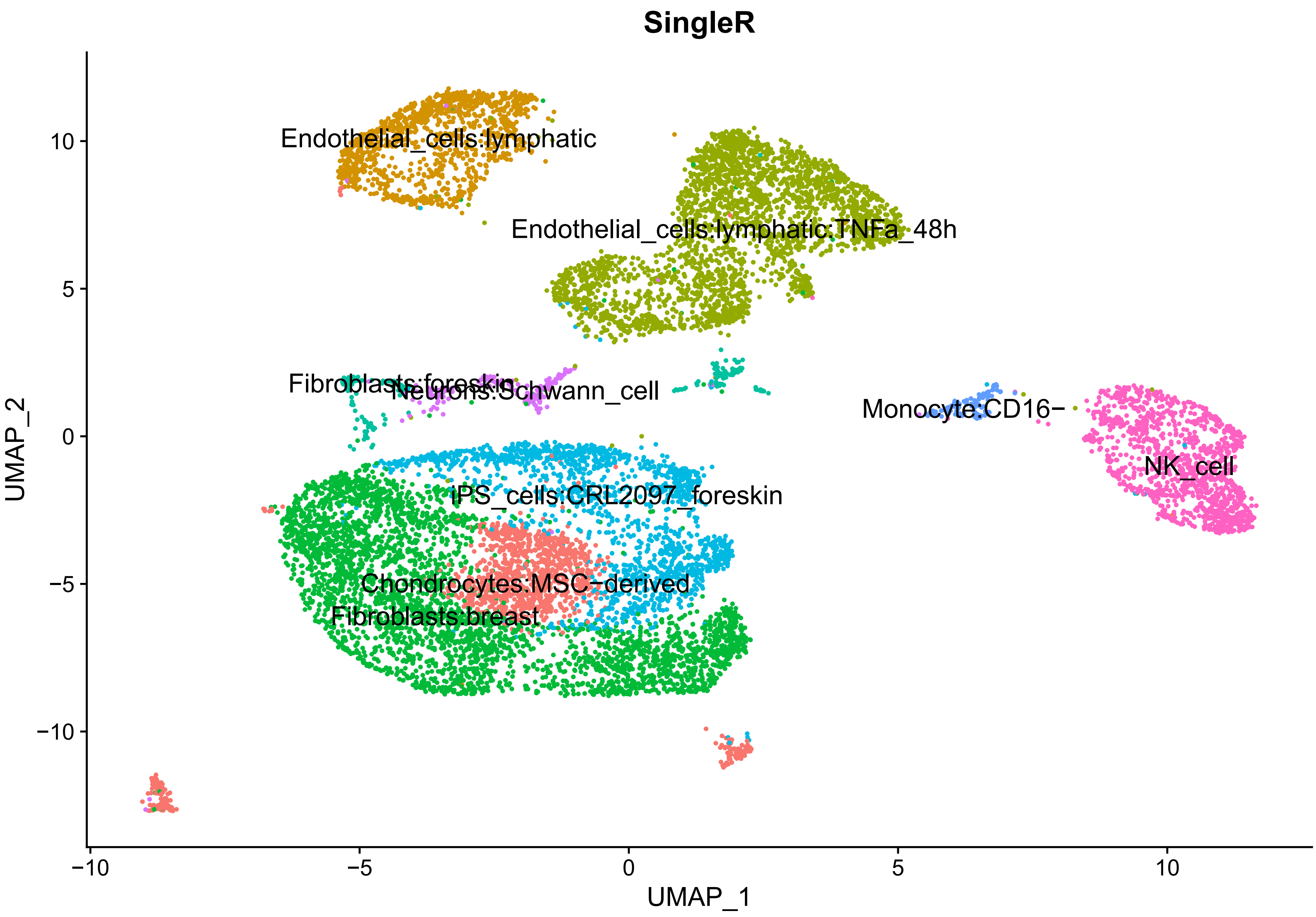

Supporting information figure S2 Scatter plot exhibiting the cell types within yak ovary annotated by SingleR package
