# Supplementary material for: A single‐cell transcriptomic atlas characterizes cell types and their molecular features in yak ovarian cortex
Source: FASEB J. 2022 Dec 17;37(1):e22718. doi: 10.1096/fj.202201176RR (PMC13281852; doi:10.1096/fj.202201176RR)
Supplement: Supplementary file 4 — Figure S4. [file FSB2-37-e22718-s006.pdf]

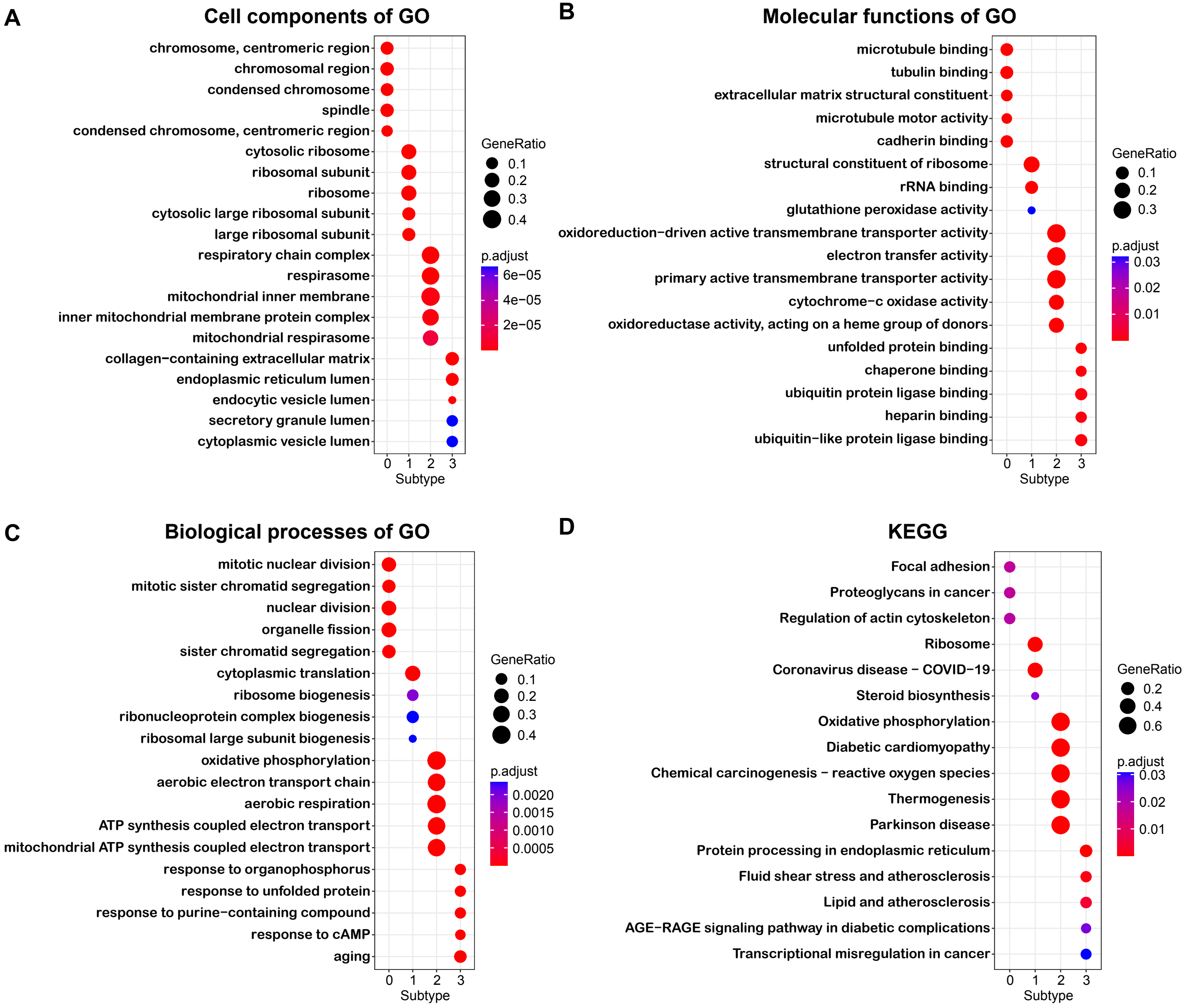

Supporting information figure S4 Gene ontology (GO) and Kyoto encyclopedia of genes and genomes (KEGG) enrichment for yak oocyte subtypes. (A-C) Bubble plots demonstrating gene ontology (GO) terms enriched for oocyte subtypes, including cell components (A), molecular functions (B) and biological processes (C). (D) Bubble plot exhibiting Kyoto encyclopedia of genes and genomes (KEGG) pathway terms enriched for oocyte subtypes.
